# In Situ Synthesis of a Tumor Microenvironment‐Activated Radiosensitizing Cu_2‐x_S/LDH Probe for Photoacoustic Imaging‐Guided Radiotherapy

**DOI:** 10.1002/advs.202522370

**Published:** 2026-01-25

**Authors:** Kang Zhu, Qiaoqiao Wei, Ying Wu, Qingqing Li, Luntao Liu, Chunxiang Mo, Chuqiao Liu, Lichao Su, Jibin Song

**Affiliations:** ^1^ State Key Laboratory of Chemical Resource Engineering College of Chemistry Beijing University of Chemical Technology Beijing P. R. China; ^2^ College of Chemistry Fuzhou University Fuzhou P. R. China

**Keywords:** photoacoustic imaging, radiotherapy, responsive bioimaging, X‐ray

## Abstract

The efficient delivery of theranostic nanoprobes to tumor sites remains a major challenge, often hindering precise tumor imaging and effective treatment. In this study, we present a novel strategy for the in situ generation of imaging and radiosensitizing nanoprobes inside tumors, enabling responsive photoacoustic imaging and targeted radiotherapy. The designed organic–inorganic hybrid nanoprobe is composed of a copper‐based layered double hydroxide (Cu‐LDH) intercalated with the near‐infrared organic dye IR‐806, serving as a photoacoustic imaging agent (designated as CAL‐IR). Upon exposure to hydrogen sulfide (H_2_S) in colorectal tumor microenvironments, the nanoprobe undergoes activation and transforms in situ into copper sulfide (Cu_2__−__x_S) nanoparticles. The newly formed Cu_2__−__x_S/LDH heterojunction nanostructure exhibits significantly enhanced near‐infrared absorption, while concurrently promoting radiotherapy efficacy through multiple mechanisms: consumption of endogenous H_2_S, reduction of X‐ray attenuation, and intrinsic radiosensitization. This unique system allows high‐contrast photoacoustic imaging and, consequently, achieves improved radiotherapy outcomes in mouse models of colorectal cancer, with minimal off‐target effects. These results underscore the promising clinical potential of CAL‐IR for precision tumor imaging and enhanced radiotherapy.

## Graphical abstract

We reported a new kind of organic‐inorganic composite nanomaterial with excellent PA imaging performance after the in situ activation by hydrogen sulfide in colorectal cancer tissues, which was used for precise radiotherapy of colorectal cancer. It shows a great application potential for early diagnosis and treatment of tumor.

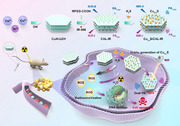

## 1 Introduction

Colorectal cancer is one of the most common cancers globally, ranking as the second leading cause of cancer‐related death, posing a significant threat to human health [1, 2, 3]. Screening, early diagnosis, and treatment of colorectal cancer can effectively reduce mortality rates [4, 5]. Hydrogen sulfide (H_2_S) is a crucial gas signaling within cells that is closely associated with various physiological and pathological processes [6, 7]. The high expression of the enzymes cystathionine β‐synthase and cystathionine γ‐lyase in colorectal cancer tissues results in abundant production of H_2_S(0.3–3.4 mm) [8, 9], which is involved in the progression and metastasis of this tumor. The high concentration of endogenous H_2_S in colorectal cancer forms a unique tumor microenvironment (TME) compared to other TMEs characterized by the presence of factors such as weak acidity, hypoxia, high glutathione, and hydrogen peroxide. Therefore, H_2_S represents a potential target for the precise diagnosis and treatment of colorectal cancer. The design and synthesis of an optical nano‐probe with high sensitivity, low signal‐to‐noise ratio, and high selectivity for H_2_S at the lesion site are crucial aspect to allow a precise diagnosis and prognosis of colorectal cancer.

Photoacoustic imaging (PAI), with its features including high spatial resolution, sensitivity, non‐invasiveness, real‐time imaging, and deep tissue penetration, has emerged as a promising biomedical imaging technique [10, 11]. An increasing number of PA contrast agents have been recently designed and developed by researchers for disease diagnosis and treatment in the biomedical field. However, most of them are of the “always on” type, and have a low signal‐to‐noise ratio and poor specificity, both of which leading to diagnostic errors [12, 13, 14]. Activatable PA contrast agents [15, 16, 17, 18] specifically respond to biomarkers in the pathological microenvironment of a diseased tissue, such as metal ions [19, 20], enzymes [21, 22], reactive oxygen species (ROS) [23], and other bioactive small molecules [24], enabling the specific imaging of a diseased tissue [25, 26]. This helps to reduce background signals, improve the signal‐to‐noise ratio, and reduce the risk of false positive diagnosis [27]. Therefore, the design and construction of activatable PA imaging contrast agents is one of the main trends in current research.

Radiotherapy possesses unique therapeutic advantages due to its tissue‐depth‐independent nature [7, 28, 29]. Researchers have explored and developed a range of physical, chemical, and biological approaches to alter and modulate tumor cell sensitivity to radiation thanks to the advancement in tumor molecular biology research [30]. For instance, multifunctional nano‐probes have been used as radiosensitizers or dose enhancers to increase the response of malignant tissues to radiotherapy [31]. These probes include nanoparticles with noble metal materials of high atomic number such as gold, silver, and gadolinium [32, 33], which can effectively deposit X‐ray energy due to their high photoabsorption cross‐section [34, 35]. Additionally, these metals interact with radiation, generating secondary electrons such as photoelectrons, Auger electrons, and Compton electrons that directly damage DNA and induce the formation of ROS through the interaction with water, further sensitizing tumor cells to radiation [36]. Semiconductor materials like titanium dioxide (TiO_2_) and zinc oxide (ZnO) have also been reported [37, 38] as able to produce electrons (e^−^) and holes (h^+^) under high‐energy radiation excitation, with holes exhibiting strong oxidative ability by abstracting electrons from water, generating highly reactive hydroxyl radicals (·OH) that damage tumor cells. Furthermore, certain transition metal chalcogenides such as tungsten disulfide (WS_2_), bismuth selenide (Bi_2_Se_3_), bismuth sulfide (Bi_2_S_3_), copper sulfide (CuS), and copper bismuth sulfide (Cu_3_BiS_3_) are potential radiosensitizers due to their high near‐infrared photothermal conversion efficiency and significant X‐ray attenuation ability, thus promising enhancers of the efficacy of radiotherapy [39]. However, ROS generated during radiotherapy are scavenged by the high amount of reducing substances present in the TME, such as glutathione and H_2_S, thereby reducing the cytotoxic effect of radiotherapy on cancer cells [40]. The introduction of nano‐probes that absorb and clear endogenous reducing substances in cancer cells alters the TME, thus increasing the radiosensitization effect of radiotherapy and inhibiting the repair mechanisms of tumor cells [41, 42].

**FIGURE 1:**
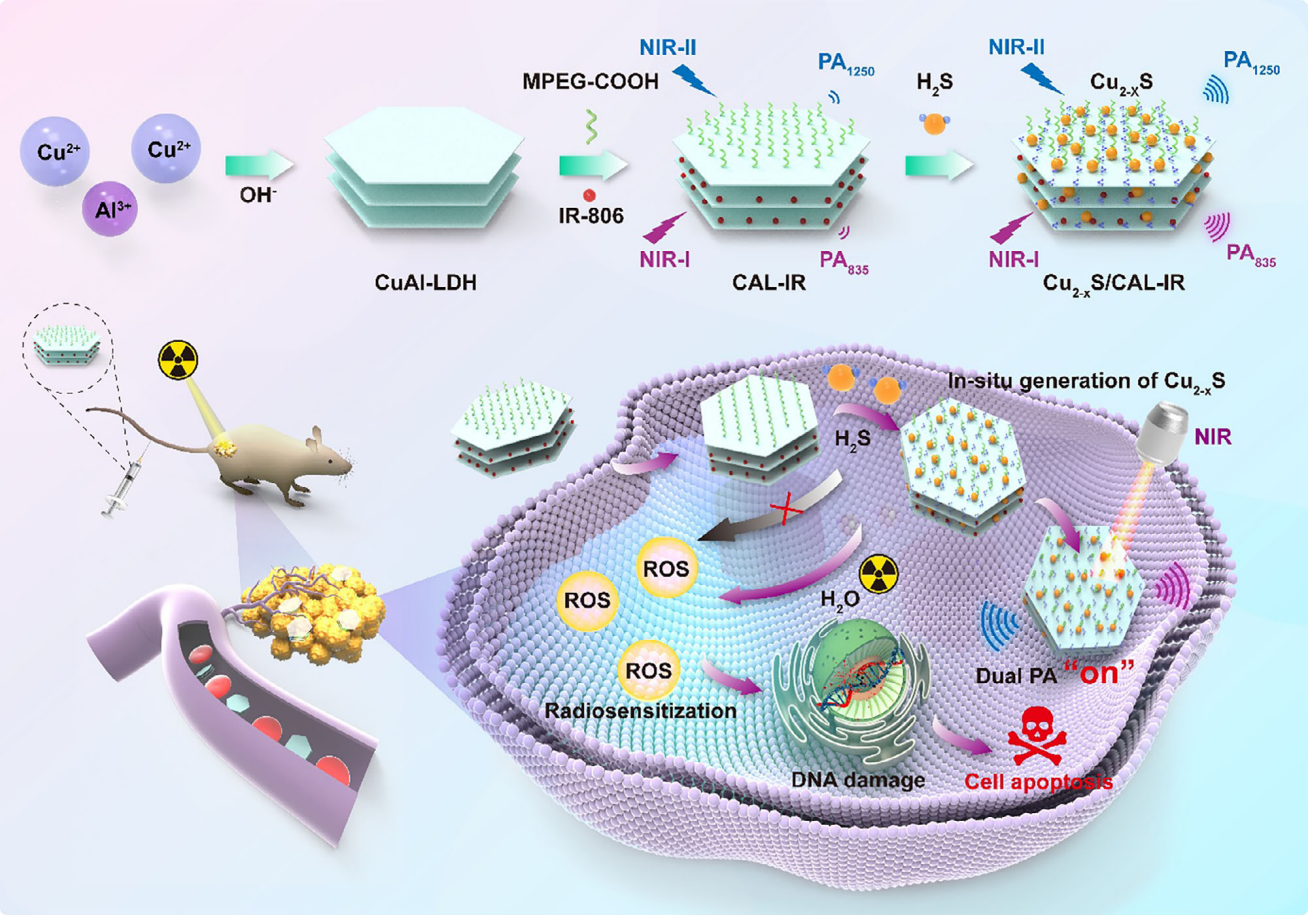
Schematic illustration of the preparation of 2D layered nanosheet CAL‐IR and its application in in situ activated PA imaging and radiotherapy of tumors. IR‐806 is a small near‐infrared dye molecule that is intercalated in Cu‐LDH, forming two different aggregation states. The as‐prepared nano‐CAL‐IR reacts with endogenous H_2_S to generate Cu_2‐x_S nanoparticles in the tumor region, resulting in PA signal changes and radiosensitizition performances. The combination with radiosensitizition therapy allows precise imaging and treatment of colon cancer.

According to these factors, a new kind of nanoprobe with TME‐responsive PA imaging preformances was developed for colon cancer tissue imaging and improving X‐ray radiosensitizition therapy. The 2D layered nanosheet‐based nanoprobe of copper‐based layered double hydroxide (Cu‐LDH) was initially prepared as precursors using a co‐precipitation method (Figure 1). Subsequently, IR‐806 was loaded onto the Cu‐LDH layer through anion exchange/adsorption and was subjected to Polyethylene glycol (PEG) surface modification to obtain a nanoprobe, named CAL‐IR. This probe was specifically responsive to H_2_S abundant in colon cancer cells, leading to the in situ generation of copper sulfide (Cu_2‐x_S) quantum dots. This, in turn, the increased optical absorption in NIR‐II, enabling the PA imaging with increased tissue penetration depth (PA_1250_). Moreover, the in situ generation of Cu_2‐x_S/LDH heterojunction altered the microstructure and electrochemical properties of the Cu‐LDH layer, inducing a transition in the aggregation mode of IR‐806 from its original J‐type and H‐type to amorphous stacking. Consequently, the absorption of CAL‐IR at 835 nm was increased, leading to a significant improvement in the PA signal of the probe in the NIR‐I window (PA_835_). Both PA signals were concurrently activated by H_2_S stimulation, enabling the precise imaging of colon lesions and minimizing the risk of false positive diagnosis. Compared to a single photoacoustic signal, the dual‐signal approach exhibits greater robustness against experimental variations and superior interference resistance, yielding more accurate and reliable measurements. Subsequently, its efficacy in radiation therapy was discovered through research on CAL‐IR‐enhanced X‐ray radiation therapy for colon cancer. Thus, this study provides new insights into a precise diagnosis of colon cancer and improvement of X‐ray radiaosensitizition therapy.

## 2 Results and Discussion

### 2.1 Synthesis and Characterization of CAL‐IR

**FIGURE 2:**
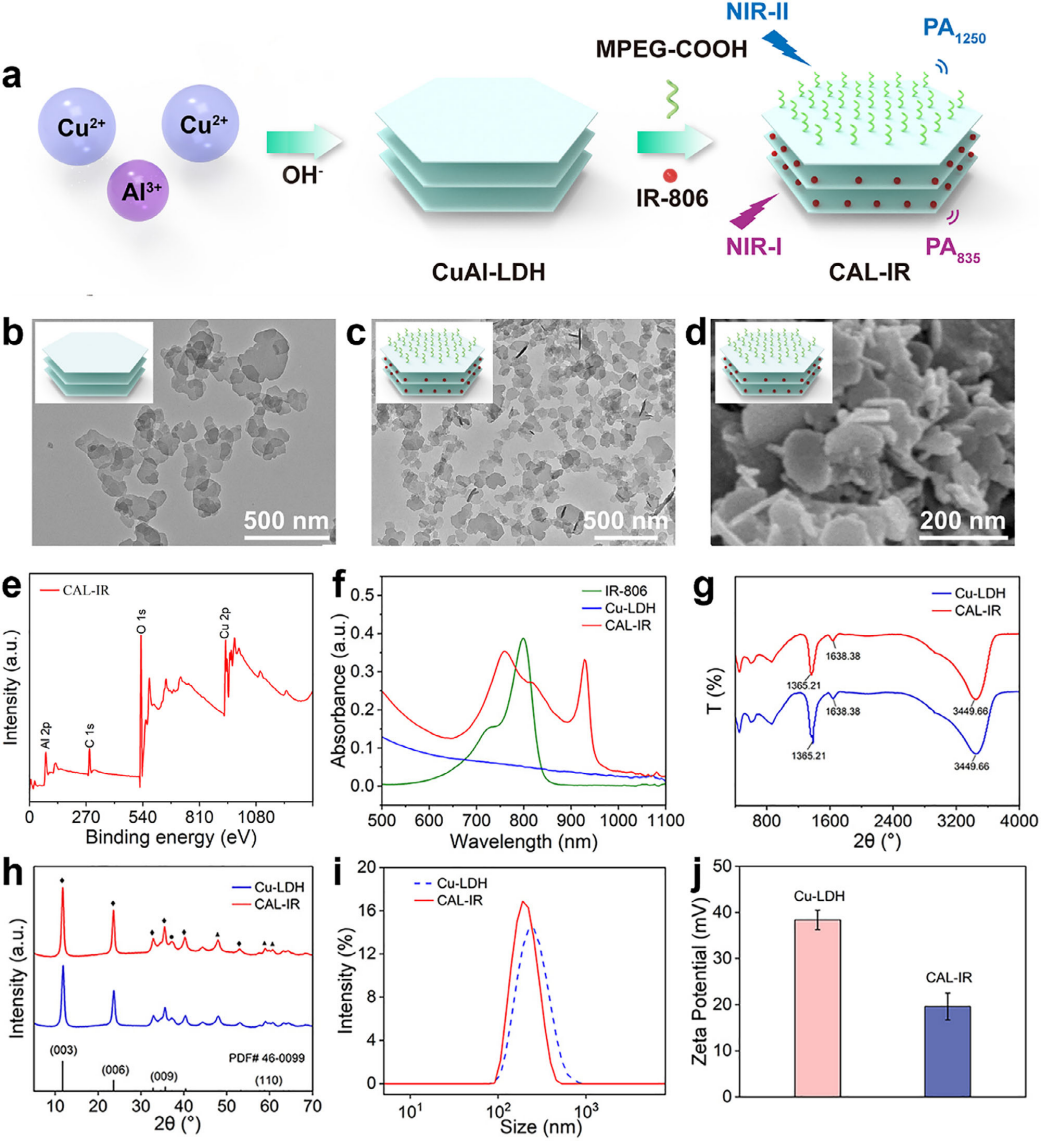
(a) Schematic illustration of the preparation of 2D layered nanosheet CAL‐IR. TEM images of (b) Cu‐LDH and (c) CAL‐IR. (d) SEM images of CAL‐IR. (e) X‐ray photoelectron spectroscopy (XPS) analysis of CAL‐IR. (f) UV–vis–NIR spectra of IR‐806 (green), Cu‐LDH (blue) and CAL‐IR (red). (g) FT‐NIR spectrum, (h) XRD, (i) particle size, and (j) zeta potential of Cu‐LDH and CAL‐IR. (*n* = 3).

CuAl‐LDH nanosheets (Cu‐LDH) were first synthesized using a typical procedure through a co‐precipitation method. IR‐806 was then intercalated into the Cu‐LDH interlayers using ion exchange, followed by modification with COOH‐PEG to enhance the biocompatibility of CuAl‐LDH nanosheets (Figure 2a). Transmission electron microscopy (TEM) and scanning electron microscope (SEM) images confirmed the lamellar structure of CAL‐IR, with an average size of approximately 150 nm (Figure 2b–d). The successful preparation of Cu‐LDH should be assessed before intercalating IR‐806. The Element surface scanning (EDS) analysis confirmed the presence of Cu and Al elements (Figure S1). X‐ray photoelectron spectroscopy (XPS) analysis (Figure S2) showed that the characteristic peaks at 77.3 and 934.3 eV corresponded to the Cu 2p and Al 2p orbitals, respectively, while the peak at 531.3 eV belonged to the O 1s orbital. The characteristic peak of C 1s appeared at 284.8 eV in the material. These data were in agreement with the XPS spectra reported in the literature for CuAl‐LDH, further confirming the successful synthesis of the Cu‐LDH nanosheet precursor [43]. The XPS spectra of CAL‐IR and Cu‐LDH were basically consistent after inserting IR‐806 into Cu‐LDH (Figure 2e). Moreover, the UV–vis absorption spectrum (Figure 2f) showed minimal absorption in the near‐infrared region, particularly in the NIR‐II, of the precursor Cu‐LDH. The maximum absorption peak of IR‐806 was around 800 nm. Interestingly, a significant change in the position of the maximum absorption peak was observed after IR‐806 was loaded onto the 2D Cu‐LDH nanosheets. This was attributed to the formation of two distinct aggregation states of IR‐806 on the Cu‐LDH nanosheets: a broad redshift absorption band at 925 nm, indicating the formation of J‐type aggregates appeared simultaneously with a clear blueshift from 800 to 760 nm attributed to the face‐to‐face *π*–*π* stacking of H‐type aggregates [44]. The formation of these two different aggregation states would greatly impact their photothermal properties, providing effective inspiration for future research. Fourier‐transform infrared (FT‐IR) spectra revealed that the band near 3449.66 cm^−1^ in the Cu‐LDH sample originated from the stretching vibration of hydroxide and interlayer water molecules. The band near 1638.38 cm^−1^ was related to the deformation vibration of water molecules. The characteristic peak at 1365.21 cm^−1^ was attributed to the anti‐symmetric stretching N─O vibration band of NO‐3, indicating the successful intercalation of NO^3−^ and successful preparation of the Cu‐LDH precursor. The positions of absorption bands in the spectrum of CAL‐IR were generally similar compared with the Cu‐LDH precursor sample, but the absorption band belonging to NO‐3 at 1365.21 cm^−1^ was weaker, indicating that some NO^3−^ was replaced by IR‐806 molecules, fully demonstrating the successful loading of IR‐806 onto Cu‐LDH (Figure 2g). Subsequently, the X‐ray diffraction (XRD) patterns of Cu‐LDH and CAL‐IR showed a series of sharp characteristic diffraction peaks corresponding to the (003), (006), (009), and (110) crystal planes of the layered structure of hydrotalcite, with sharp and good symmetry, suggesting the formation of highly crystalline copper‐aluminum hydrotalcite. Additionally, a small amount of CuO and CuAlO_2_ was observed, which was a normal phenomenon due to the strong Jahn–Teller effect of copper [45]. The XRD patterns showed little change before and after intercalation, indicating that it had little effect on the layered structure and crystallinity of Cu‐LDH (Figure 2h).

The results of dynamic light scattering showed the average hydrodynamic size of the precursor Cu‐LDH, as well as of the composite nanoparticle CAL‐IR after IR‐806 intercalation, and PEG surface modification in ultrapure water (Figure 2i). The hydrodynamic size decreased after intercalation and modification, indicating that the modification of PEG surface improved the dispersion of composite nanoparticles in water. Furthermore, the changes in zeta potential of intermediates and products in different preparation steps were assessed; the zeta potential of the Cu‐LDH precursor was 38.4 mV, which decreased to 19.6 mV after IR‐806 intercalation and PEG modification, indicating the successful intercalation and PEG modification (Figure 2j).

### 2.2 Response of the Nanoprobe CAL‐IR Changes to CuS Triggered by H_2_S in Vitro

**FIGURE 3:**
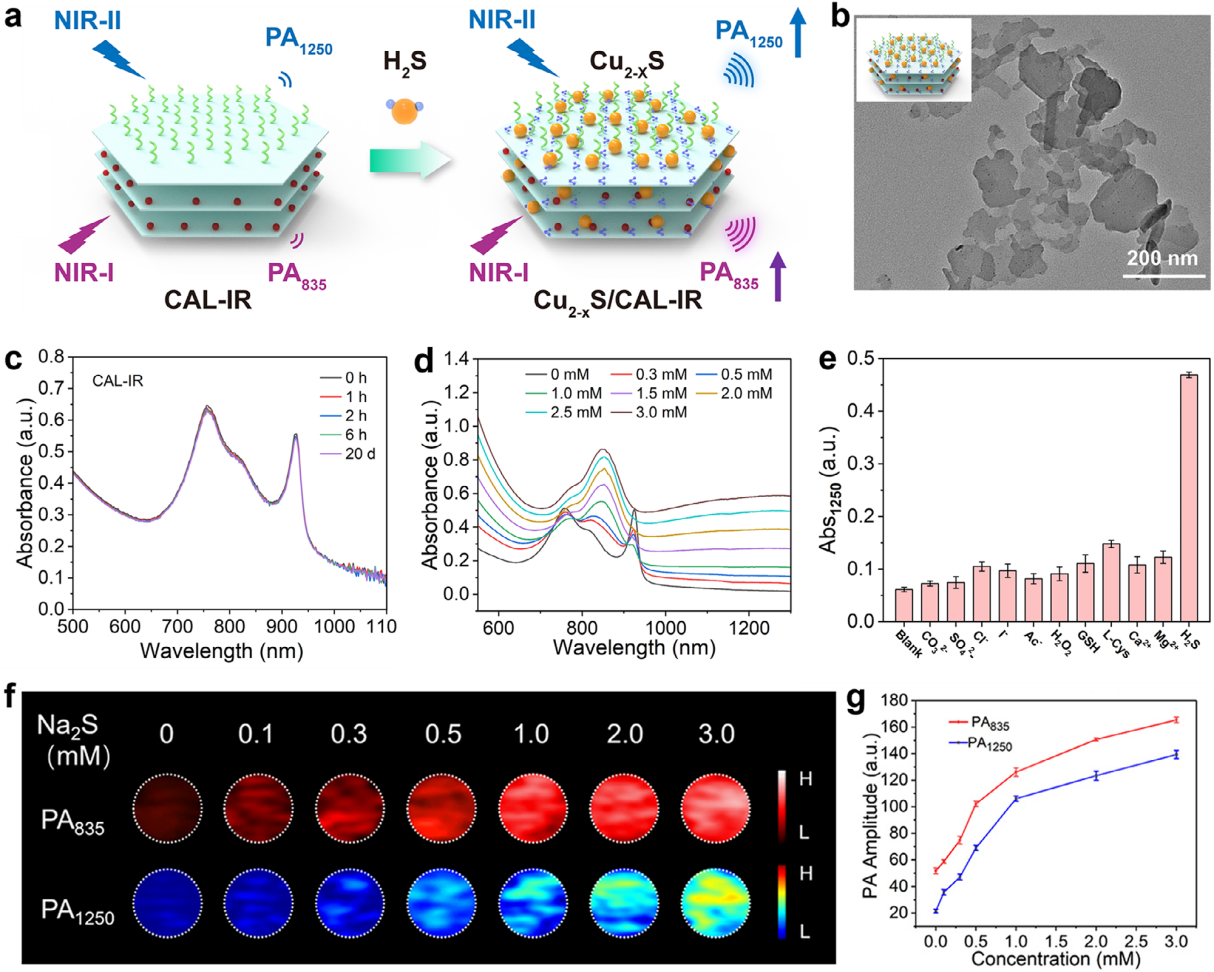
(a) Schematic diagram of CAL‐IR responding to Na_2_S to generate Cu_2‐x_S/CAL‐IR. (b) TEM image of the obtained Cu_2‐x_S/CAL‐IR. (c) UV–vis absorption spectra of CAL‐IR over time. (d) UV–vis–NIR spectra of CAL‐IR at different concentrations of Na_2_S (0–3.0 mm). (e) The absorbance at 1250 nm of CAL‐IR in reaction to different signaling molecules. (f) NIR‐I and NIR‐II PA images of CAL‐IR after the incubation with different concentrations of Na_2_S (0–3 mm), and (g) the corresponding PA intensity (*n* = 3).

To study the responsive performance of the nanoprobe, Na_2_S was chosen as the precursor of H_2_S, which reacted with the composite nanoprobes CAL‐IR to produce Cu_2‐x_S/CAL‐IR to assess the properties of CAL‐IR nanoprobes toward hydrogen sulfide (Figure 3a). After reacting with H_2_S, the nanosheets were observed to have grown a large number of spherical particles, as shown in the TEM image (Figure 3b). Additionally, the number of spherical particles growing on the nanosheets showed an upward trend as the reaction time and Na_2_S concentration increased (Figures S3 and S4). Previous studies indicated that cyanine dyes such as ICG and IR‐806 have low stability in aqueous solutions and under light conditions [46]. These dyes contain easily oxidizable groups prone to react with oxygen in the air, leading to oxidation and loss of color. Some cyanine dyes are also sensitive to changes in environmental pH; the stability of the dye is affected when it changes significantly, and decomposition is prone to occur. Additionally, structural damage leads to a decrease in photothermal conversion efficiency. Therefore, it is essential to examine and verify the stability of materials. Both IR‐806 aqueous solution and CAL‐IR water dispersion samples were stored in the dark at 4 °C, and their changes in UV–visible absorption spectra over time were tested (Figure 3c; Figure S5). The results showed that the maximum absorption peak intensity of IR‐806 existing as free monomers in the aqueous solution gradually decreased with increasing storage time. The decrease approached 70% after 10 h of storage, indicating the poor stability of the IR‐806 aqueous solution. In contrast, the absorption peak of the CAL‐IR water dispersion sample obtained after intercalation remained almost unchanged over the entire test wavelength range during the increasing storage time. The absorption intensity remained basically unchanged even after storage at room temperature for 20 days, indicating the protective and stabilizing effect of the layered structure on small dye molecules, which was beneficial for the storage and further practical application of the material. UV–vis absorption spectra showed the changes in absorption intensity in the NIR‐I and NIR‐II windows after the incubation of CAL‐IR probes with different concentrations of Na_2_S (0–3.0 mm) (Figure 3d). Particular attention was given to the absorption at 1250 nm in the NIR‐II window. The Cu^2+^ on the layer of CAL‐IR nanoprobes reacted with S^2−^ ions ionized in the Na_2_S solution to generate Cu_2‐x_S quantum dots; the increase in H_2_S (Na_2_S) concentration led to more Cu_2‐x_S production, thus leading to a gradual increase in the absorption observed at 1250 nm in the spectra. Concurrently, the formation of heterojunction Cu_2‐x_S quantum dots affected the microstructure and charge properties of the Cu‐LDH layer, causing a rearrangement of interlayer small molecules. The originally face‐to‐face parallel stacking H‐type aggregates (blueshift, 760 nm) and misaligned parallel stacking J‐type aggregates (redshift, 925 nm) were transformed into an amorphous arrangement. Consequently, the absorption peaks corresponding to the first two aggregates gradually weakened, while the absorption peak at 835 nm corresponding to the amorphous arrangement gradually strengthened. The overall absorption values shifted across the entire wavelength range due to the generation of more Cu_2‐x_S, which had a broader absorption peak in the near‐infrared region. In addition, only H_2_S significantly induced a change in absorbance at 1250 nm compared with other signaling molecules, demonstrating that CAL‐IR had excellent selectivity and specificity (Figure 3e).

In the subsequent experiments, PA properties of CAL‐IR nanoparticles were investigated in response to different concentrations of H_2_S by measuring changes in the PA signals (Figure 3f,g). Wavelengths of 835 and 1250 nm were selected for PA imaging tests in vitro, as shown in Figure 2d,e. The PA intensity at both wavelength positions showed a positive correlation with the concentration of H_2_S, consistent with the trends observed in their absorbance changes. These test results demonstrated the successful construction of a PA imaging nanoprobe responsive to H_2_S through a “turn‐on” strategy at dual wavelengths in vitro, offering a new experimental foundation for further cellular and in vivo imaging application of this probe.

### 2.3 In Vitro PA Imaging and Therapeutic Effects of CAL‐IR Nanoparticles on CT26 Cells

**FIGURE 4:**
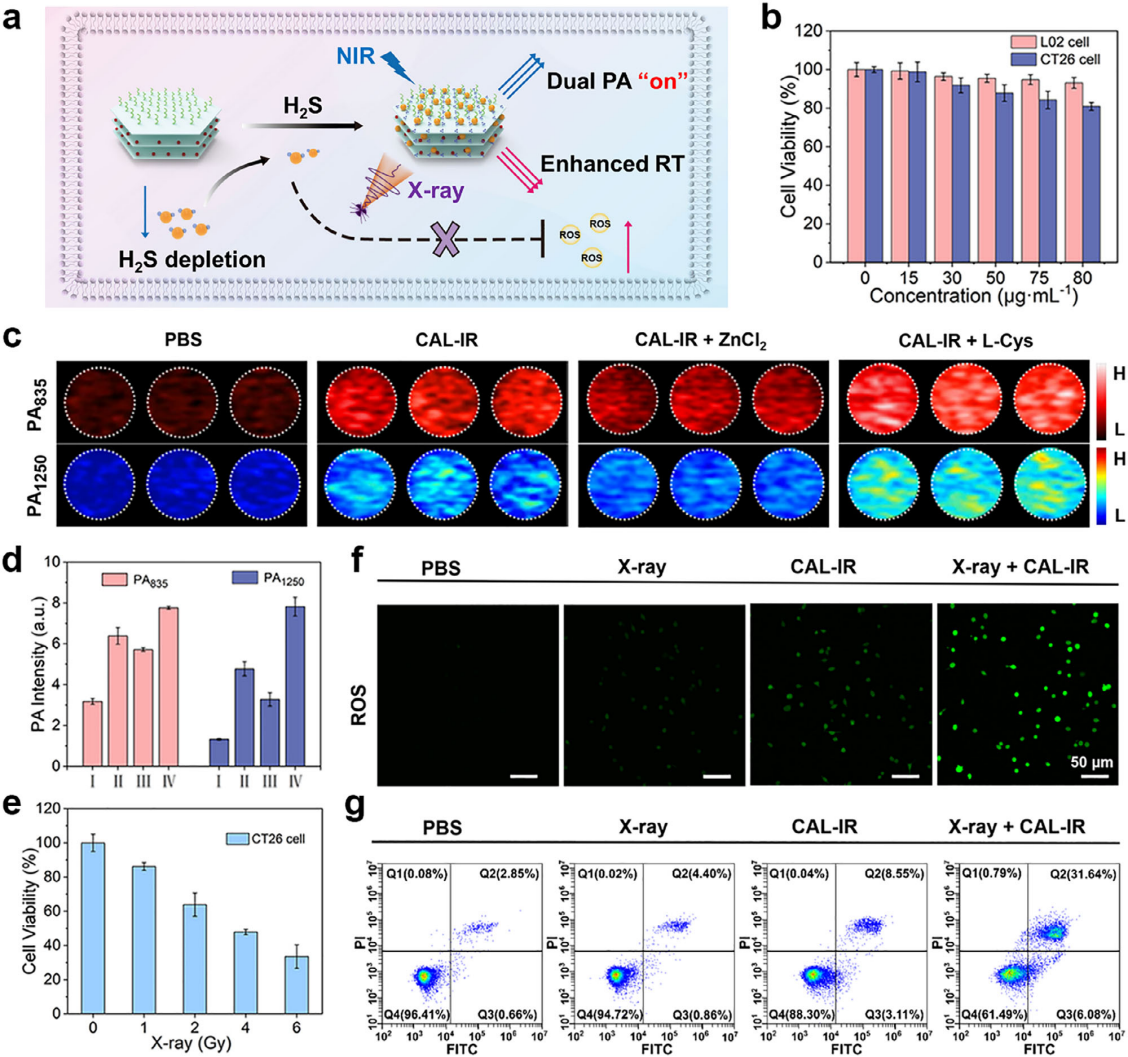
(a) Schematic illustration of CAL‐IR for imaging and therapy in vitro. (b) Relative cell viability of L02 and CT26 cells after the treatment with different concentrations of CAL‐IR. (c) and (d) NIR‐I and NIR‐II PA images of CT26 cells after different treatments. (I: PBS; II: CAL‐IR, III: CAL‐IR + ZnCl_2_; IV: CAL‐IR + L‐Cys) and the corresponding PA intensity of CT26 cells in different groups. (*n* = 3) (e) Viability of CT26 cells treated with CAL‐IR and irradiated with different doses of X‐ray. (f) Confocal fluorescence microscope images of CT26 cells treated with different methods. (X‐ray dose: 4 Gy; Scale bar: 50 µm). (g) Contour diagram of FITC‐Annexin V/PI by flow cytometry and of CT26 cells after different treatments. X‐ray dose: 4 Gy; Q1 represents necrotic cells; Q2 shows non‐viable late apoptosis/necrosis cells, which are positive for FITC‐Annexin V binding and PI uptake; Q3 represents apoptotic cells, FITC‐Annexin V positive, and PI negative; Q4 shows viable cells without PI and negative for FITC‐Annexin V binding.

The composite nanoprobes CAL‐IR were successfully synthesized using our previous experiments, and their responsiveness to H_2_S was confirmed in vitro, as well as their ability to generate dual‐wavelength PA signals in both the NIR‐I and NIR‐II regions. DLS also confirmed that CAL‐IR surfaces were positively charged, which allowed the targeting and accumulation of the probe in the tumor. Based on these theoretical and empirical findings, the changes in PA imaging signals induced by H_2_S responsiveness of CAL‐IR in colorectal cancer cells CT26 were assessed, as well as their performance in X‐ray radiation therapy (Figure 4a). The cytotoxicity of the composite nanoprobes CAL‐IR was assessed on normal human liver cells L02 cells before performing cellular PA imaging. The results indicated that the viability of L02 remained significantly high as the concentration of CAL‐IR probes increased gradually from 0 to 80 µg/mL, demonstrating the high biocompatibility of the probe with normal cells. However, CT26 cell viability remained above 80% at the concentration of 80 µg/mL of the material, although a slight decrease was observed with increasing probe concentration. These results suggested that higher concentrations of the material exerted slight toxicity to CT26 cells (Figure 4b).

In the following, the cells were divided into four groups (I: PBS group; II: CAL‐IR group, III: CAL‐IR + ZnCl_2_ group; IV: CAL‐IR + L‐Cys group) and subjected to different methods (with L‐Cys as an enhancer of intracellular H_2_S and ZnCl_2_ as an inhibitor), followed by the collection of cells for PA imaging using a PA imaging instrument to demonstrate that the intracellular imaging of CAL‐IR was triggered by H_2_S and that the strength of the imaging signal was correlated with H_2_S content (Figure 4c,d). Only the PBS control group of CT26 cells showed no significant PA signal among these four groups. The groups incubated with the probe exhibited prominent PA signals at both 835 and 1250 nm wavelengths. The PA signals at both wavelengths were slightly reduced compared to the probe group in the inhibition group treated with ZnCl_2_ and the probe, where some intracellular H_2_S was cleared. Furthermore, the response was more pronounced in the enhancement group treated with the H_2_S enhancer L‐Cys followed by probe incubation due to the increased intracellular H_2_S levels, resulting in significantly enhanced PA signals at both 835 and 1250 nm wavelengths compared to the probe group. These results of H_2_S‐responsive PA imaging demonstrated that the composite nanoprobes CAL‐IR was activated by different concentrations of H_2_S in the cells, simultaneously opening up PA signals at both the NIR‐I window (835 nm) and NIR‐II window (1250 nm). This evidence highlighted the excellent intracellular activatable PA imaging ability of CAL‐IR, providing support for its subsequent application in endogenous H_2_S‐responsive PA imaging in solid tumors.

The cytotoxicity and biocompatibility experiments revealed that cell viability was slightly above 80% at a probe concentration of 80 µg/mL when CAL‐IR was incubated with CT26 cells alone. Consequently, this concentration was selected to investigate the effects of different X‐ray doses on cancer cell death. Cells were incubated for an additional 10 h after the exposure to different doses of X‐rays and the CCK8 cytotoxicity assay was used to measure the absorbance and quantify the treatment effect on CT26 cells to evaluate the effect of radiotherapy (Figure 4e). The viability of cancer cells progressively decreased with increasing X‐ray radiation doses, confirming the radiosensitising effect of CAL‐IR on cancer cells. Cell viability showed a dose‐dependent response at 80 µg/mL CAL‐IR, achieving a 50% killing rate at a dose of 4 Gy. Thus, this dose was selected for further experimental investigations.

Cu_2‐x_S, a transition metal sulfide semiconductor material, was generated after the intracellular accumulation of the CAL‐IR probe, which has a radiosensitising effect on cancer cells. Concurrently, intracellular H_2_S was removed, disrupting the redox balance in the TME and inhibiting cancer cell repair. Additionally, the ROS produced during radiotherapy are not neutralized by H_2_S, leading to a modified TME that enhanced cellular sensitivity to radiotherapy. This resulted in the accumulation of more ROS in tumor cells, thereby increasing cell lethality. This effect was investigated by dividing the cells into four groups, which were treated by different methods, including material incubation and X‐ray radiotherapy. Next, ROS generation ability was assessed in CT26 cells. Confocal fluorescence microscopy images and quantitative analysis of fluorescence intensity (Figure 4f; Figure S6) indicated that the CAL‐IR+X‐ray experimental group showed a significant accumulation of ROS, as indicated by the stronger green fluorescence compared to that the other three control groups. This suggested that the combined treatment of cells with CAL‐IR nanoprobes and X‐ray radiotherapy promoted the production and accumulation of ROS in tumor cells, thereby demonstrating a superior radiosensitising effect. Subsequently, cell apoptosis was assessed by flow cytometry. After subjecting the four groups of CT26 cells to different treatments, dual staining with Annexin V‐FITC/PI was performed and the results were assessed by flow cytometry (Figure 4g). The total apoptotic rate (Q2 + Q3) in the X‐ray+CAL‐IR experimental group was 37.72%, significantly higher than that in the X‐ray group and the CAL‐IR group alone. The composite nanoprobe CAL‐IR exerted an excellent X‐ray radiotherapy‐induced cytotoxicity on CT26 colon cancer cells, as revealed by calcein AM and propidium iodide (PI) double staining assay (Figure S7). Co‐treatment with CAL‐IR (80 µg/mL) and Na_2_S (1 mm) slowed the rate of cell viability reduction. The addition of Na_2_S attenuated the radiosensitizing effect of CAL‐IR on cancer cells. This demonstrates that H_2_S scavenging is a key mechanism underlying radiosensitization (Figure S8).

### 2.4 In Vivo NIR‐I and NIR‐II PA Imaging of H_2_S in Subcutaneous Tumor

**FIGURE 5:**
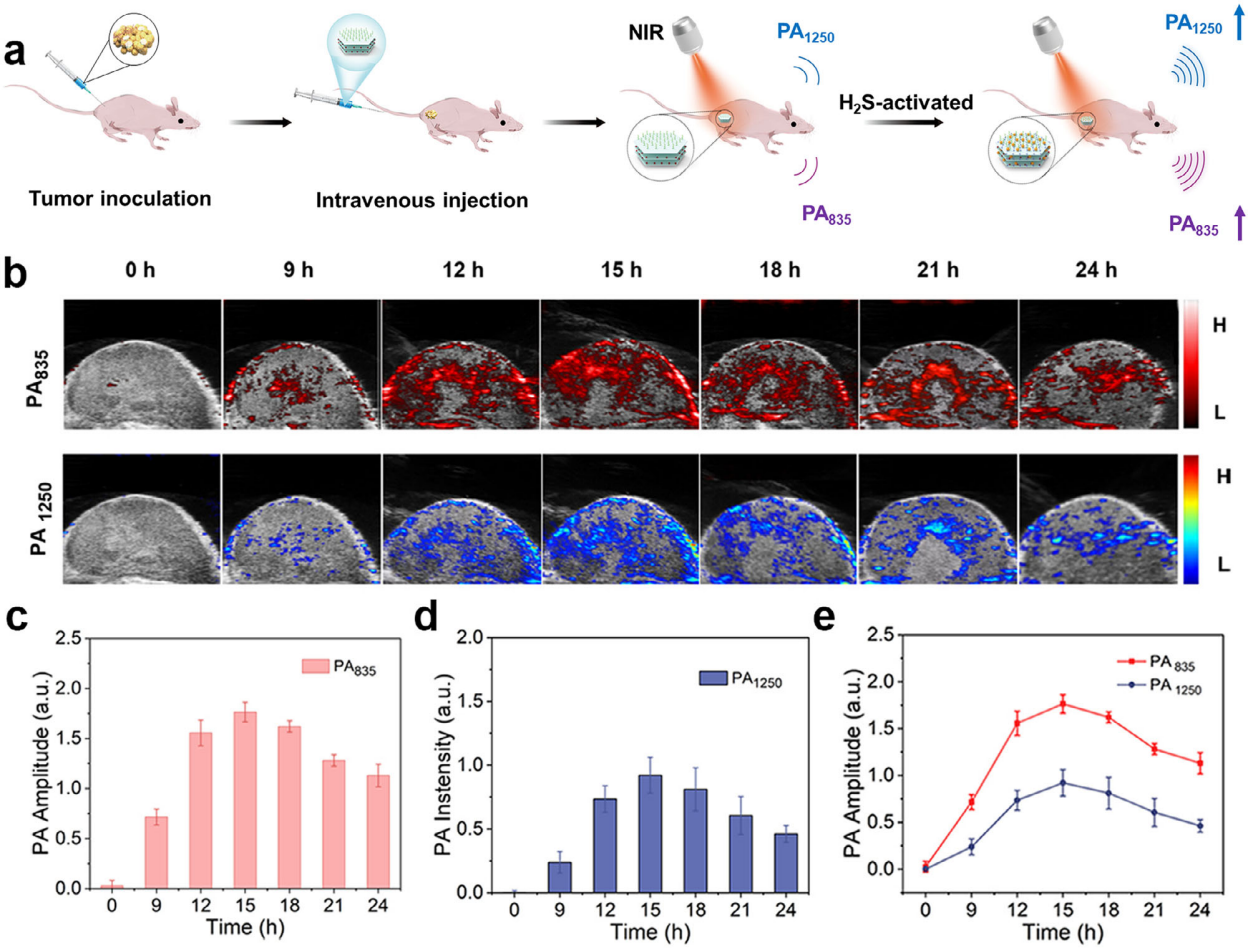
(a) Schematic diagram of the CAL‐IR nanoprobe for the detection of H_2_S and NIR‐I and NIR‐II PA imaging in subcutaneous colon cancer in mice. (b) NIR‐I and NIR‐II PA imaging at different time points after CT26 tumor‐bearing mice were treated with CAL‐IR through intravenous injection. (c), (d) and (e) NIR‐I and NIR‐II relative PA intensity of tumor‐bearing mice at different time points. (*n* = 3).

The aforementioned experiments confirmed that CAL‐IR possessed H_2_S‐activated PA imaging properties in vitro. Subsequently, its imaging ability in vivo was assessed by PA imaging tests on subcutaneous tumors in mice (Figure 5a,b). The results showed that the tumors treated with this nanoprobe emitted significant PA signals at 835 and 1250 nm, with the signal intensity peaking at 15 h and remaining detectable at 24 h (Figure 5c–e). These findings indicated that CAL‐IR nanoprobes effectively accumulated into the tumor with prolonged retention. Moreover, the results demonstrated that the nanoprobes responded to endogenous H_2_S in the tumor, successfully enabling PA imaging at both NIR‐I and NIR‐II wavelengths. Real‐time monitoring of PA images in tumor‐bearing mice facilitated the visualization of the tumor, providing significant insights for tumor imaging and therapeutic guidance.

### 2.5 In Vivo Radiotherapeutic Effect of CAL‐IR on Colon Tumors

**FIGURE 6:**
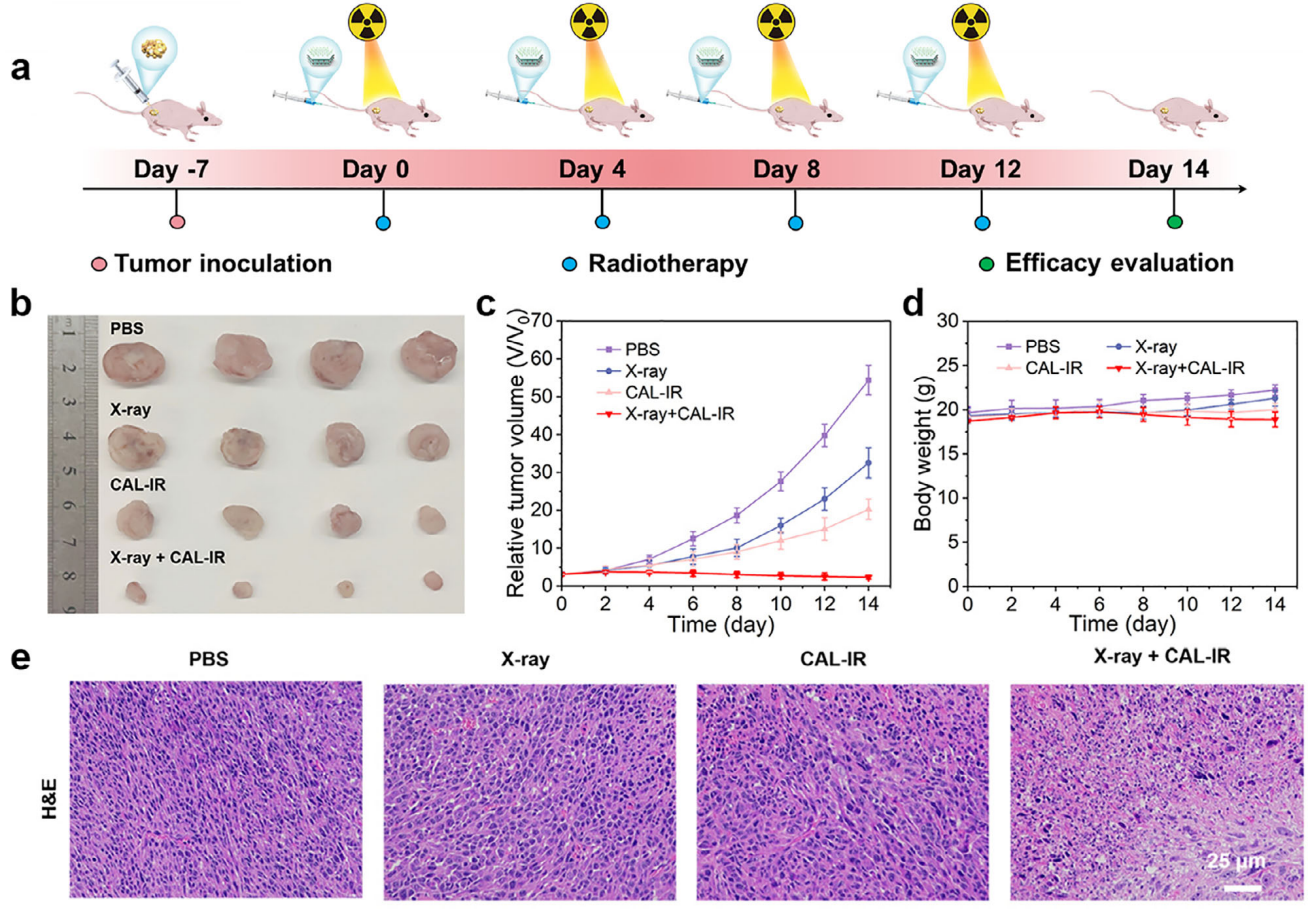
(a) Schematic diagram of the construction and treatment of CT26 tumor‐bearing mice. (b) Photographs of tumors from tumor‐bearing mice collected 14 days after receiving different treatments. (c) Tumor growth curves and (d) body weight change in different groups of tumor‐bearing mice as a function of time after different treatments. (*n* = 6) (e) H&E‐stained tumor sections collected from mice in each group after 14 days of treatment. (Scale bar: 25 µm). I: PBS group; II: X‐ray group; III: Probe group; IV: Probe + X‐ray group.

The experimental results confirmed that the composite nanoprobe CAL‐IR responded effectively to H_2_S gas in vitro and exerted significant cytotoxic effects on cancer cells under X‐ray irradiation. The in vivo responsiveness and radiotherapeutic effect of this nanoprobe was further demonstrated by anti‐tumor experiments on CT26 tumor‐bearing mice (Figure 6a–c). The PBS, X‐ray, and CAL‐IR treatment groups showed poor therapeutic outcomes. The tumors in the PBS group grew rapidly, reaching over five times their original size. In contrast, the tumor volume in the X‐ray group and the CAL‐IR group increased more slowly, probably because of the individual cytotoxic effects of X‐rays and the CAL‐IR probe on tumor cells. However, the CAL‐IR+X‐ray experimental group exhibited a significant reduction in tumor volume post‐treatment. This effect might be attributed to the in situ response of CAL‐IR with H_2_S in the colon cancer tissue, leading to the formation of Cu_2‐x_S heterostructures that weakened X‐ray attenuation, thereby increasing the radiotherapy effect. Furthermore, the clearance of H_2_S gas in the TME enhanced the effectiveness of the combined treatment, increasing the cytotoxicity of CAL‐IR on colon cancer cells. These experiments demonstrated that the composite nanoprobe CAL‐IR possessed excellent radiosensitising ability. Additionally, the health of experimental animals was crucial for the accuracy of scientific results; indeed, mouse body weight remained relatively stable throughout the study, indicating the safety and efficacy of the treatment (Figure 6d). The slight decrease in the average body weight of the experimental group mice suggested a significant tumor growth inhibition due to the treatment. The tumor was collected after treatment from each group of mice and the anti‐tumor performance of CAL‐IR was assessed using H&E staining (Figure 6e). Tumor cells in mice treated with X‐ray or CAL‐IR showed sparser nuclei compared to the PBS group. The group treated with both CAL‐IR and X‐rays showed the most pronounced tumor damage, indicating the substantial cytotoxic effect of the nanoprobe on cancer cells under radiotherapy.

**FIGURE 7:**
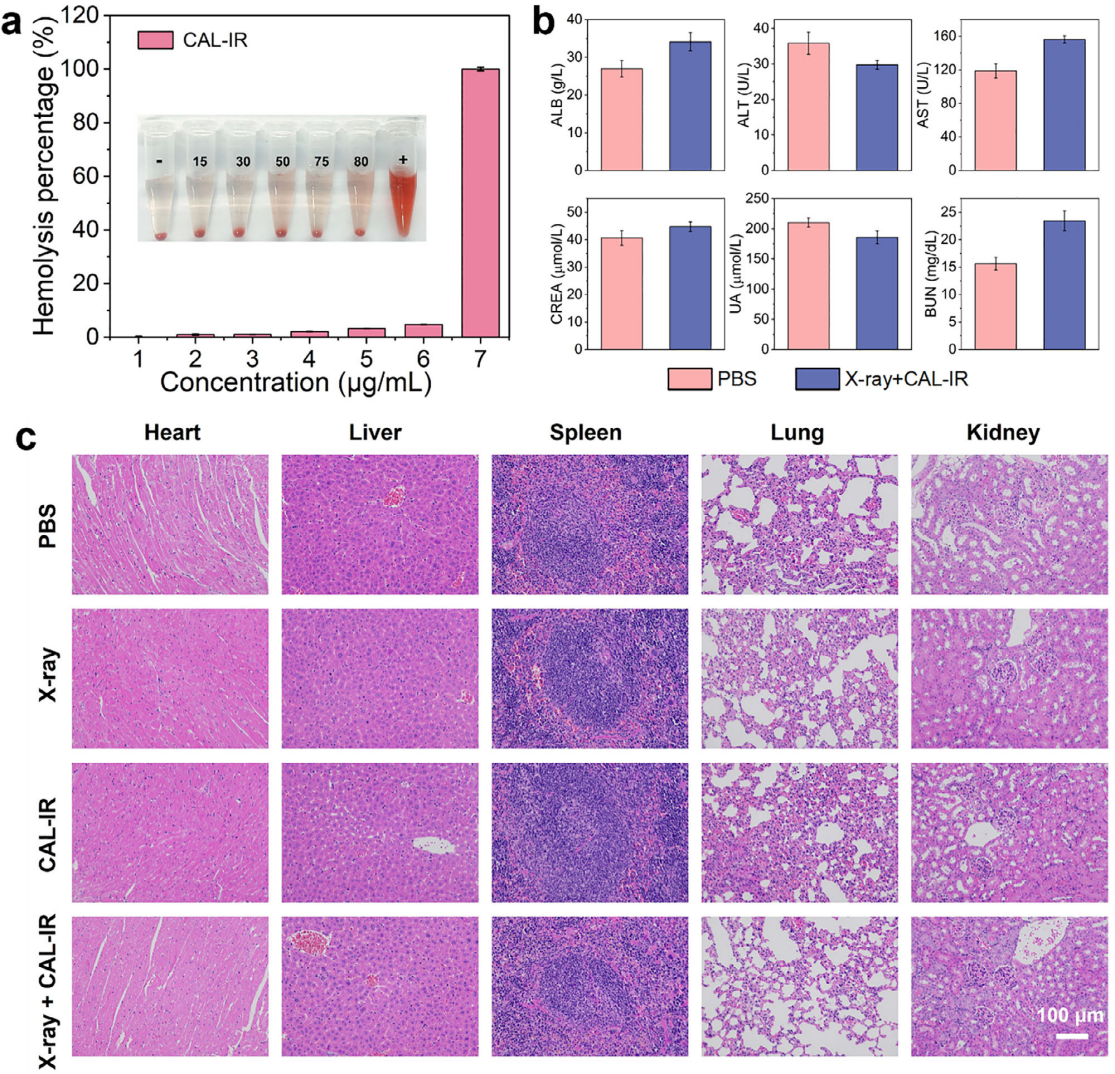
(a) The hemolysis analysis of CAL‐IR at different concentrations. Where ‐ and + denote negative and positive controls, respectively. (b) The levels of liver function indicators and kidney function indicators at PBS and X‐ray+CAL‐IR groups, respectively. (c) H&E staining of the main organs harvested from mice after different treatments (scale bar: 100 µm).

The biocompatibility of CAL‐IR was evaluated by hemolysis analysis and the results revealed that CAL‐IR possessed good hemocompatibility and induced less than 10% hemolysis even at a concentration of 80 µg/mL (Figure 7a). Blood biochemical analysis revealed that the liver, kidney function, and hematological indexes of the mice in the X‐ray+CAL‐IR treatment group were within the normal range, with no obvious toxic side effects or immune reactions (Figure 7b; Figure S9). In addition, H&E staining analysis of the main organs of mice from different treatment groups revealed no significant damage, which confirmed the biological safety of radiotherapy under CAL‐IR sensitization (Figure 7c).

## 3 Conclusion

In this work, a new kind of organic‐inorganic nanocomposite CAL‐IR was developed by intercalating the organic dye IR‐806 into Cu‐LDH, resulting in the in situ generation of a tumor‐activated near‐infrared PA imaging and radiosensitizition performances. This probe was applied to response PA image‐guided X‐ray radiotherapy on colon tumors. It specifically responded to the high amount of H_2_S in the cancer cells, generating copper sulfide (Cu_2‐x_S) nanoparticles in situ, which activated PA signals in both the NIR‐I and NIR‐II windows. The in situ formation of Cu_2‐x_S, coupled with the depletion of H_2_S, rendered CAL‐IR an effective radiosensitizer. Both in vitro and in vivo experiments demonstrated that this nanomedicine possessed a strong anti‐tumor effect with low systemic toxicity. Furthermore, CAL‐IR functioned as an effective theranostic agent: its H_2_S‐responsive dual‐PA imaging enabled accurate detection of colorectal lesions with minimal false positives, while its strong radiosensitization property significantly potentiated the antitumor effect of X‐ray therapy. Consequently, this study provided a new approach for the precise diagnosis of colorectal cancer and the enhancement of X‐ray radiotherapy.

## 4 Experimental Section

### 4.1 Synthesis of CAL‐IR

Cu‐LDH suspension was added to 10 mL ultrapure water and dissolved by ultrasound. Subsequently, 30 mL MPEG‐COOH aqueous solution was added and the solution was stirred for 5 min to ensure thorough mixing. Then, 300 µL IR‐806 1 mg/mL methanol aqueous solution (40%) was added to the suspension. This mixture was stirred for 24 h in the dark. The resulting IR‐806 intercalated and PEG‐modified LDH composite nanomaterial was collected and dried in a vacuum oven at 40°C. The final product was labeled as CAL‐IR.

### 4.2 In Vivo Radiotherapeutic Effect of CAL‐IR on Colon Cancer Subcutaneous Tumors

The peak accumulation time of the nanoprobes in the tumor was determined by in vivo PA imaging of solid tumors. This time point was selected as the optimal moment for X‐ray irradiation treatment. Mice were randomly divided into four groups when the tumors in the mice reached approximately 80 mm^3^. The PBS control group received 100 µL PBS by tail vein injection. The CAL‐IR group received 100 µL CAL‐IR probe (2 mg/mL, dispersed in PBS) by tail vein injection. The X‐ray group received 100 µL PBS by intravenous injection, followed by X‐ray irradiation of the tumor (2 Gy). The CAL‐IR+X‐ray group received 100 µL CAL‐IR probe (2 mg/mL, dispersed in PBS) by intravenous injection, followed by X‐ray irradiation of the tumor (2 Gy). The X‐ray radiotherapy was administered every 4 days for a total of four sessions, starting 15 h after the tail vein injection of the probe. The body weight and tumor volume of the mice were measured every 2 days throughout the entire experiment. All mice were humanely euthanized at the end of the 14‐day experiment, and the tumors were collected and photographed.

## Funding

National key research and development plan (NO. 2023YFB3810002), Beijing Outstanding Young Scientist Program (JWZQ20240101010), the National Natural Science Foundation of China (No. 82202239, 82373101, U21A20377, U22A20348, U24A20731), the Fundamental Research Funds for the Central Universities (PT2516, ZY2503, buctrc202235, PT2508).

## Conflicts of Interest

The authors declare no conflicts of interest.

## Supporting information




**Supporting File**: advs74027‐sup‐0001‐SuppMat.docx.

## Data Availability

The data that support the findings of this study are available in the supplementary material of this article.

## References

[1] R. L. Siegel , N. S. Wagle , A. Cercek , R. A. Smith , and A. Jemal , “Colorectal Cancer Statistics, 2023,” CA: A Cancer Journal for Clinicians 73 (2023): 233–254, 10.3322/caac.21772. 36856579

[2] H. Jia , J. Lin , D. Wang , et al., “A Mn^2+^ ‐Assisted Nanofiber‐Hydrogel Adjuvant for Simultaneous Enhancement of Humoral and Cellular Immune Responses,” Advanced Functional Materials 34 (2024): 2315442, 10.1002/adfm.202315442.

[3] F. Zhai , B. Yun , J. Ming , et al., “Non‐Invasive Diagnosis of Early Colorectal Cancerization via Amplified Sensing of MicroRNA‐21 in NIR‐II Window,” Advanced Materials 37 (2025): 2501378, 10.1002/adma.202501378. 40123304

[4] C. Xu , M. Xu , Y. Hu , et al., “Ingestible Artificial Urinary Biomarker Probes for Urine Test of Gastrointestinal Cancer,” Advanced Materials 36 (2024): 2314084, 10.1002/adma.202314084. 38446383

[5] C. Xu , X. Qin , X. Wei , et al., “A Cascade X‐Ray Energy Converting Approach Toward Radio‐Afterglow Cancer Theranostics,” Nature Nanotechnology 20 (2025): 286–295, 10.1038/s41565-024-01809-9. 39548317

[6] Z. Chen , L. Su , Y. Wu , et al., “Design and Synthesis of a Small Molecular NIR‐II Chemiluminescence Probe for in Vivo—Activated H_2_S Imaging,” Proceedings of the National Academy of Sciences 120 (2023): 2205186120, 10.1073/pnas.2205186120. PMC9974472 36787363

[7] J. Huang , L. Su , C. Xu , et al., “Molecular Radio Afterglow Probes for Cancer Radiodynamic Theranostics,” Nature Materials 22 (2023): 1421–1429, 10.1038/s41563-023-01659-1. 37667071

[8] L. An , X. Wang , X. Rui , et al., “The In Situ Sulfidation of Cu_2_O by Endogenous H_2_ S for Colon Cancer Theranostics,” Angewandte Chemie International Edition 57 (2018): 15782–15786, 10.1002/anie.201810082. 30307092

[9] D. Wang , H. Jia , H. Cao , et al., “A Dual‐Channel Ca2+ Nanomodulator Induces Intracellular Ca^2+^ Disorders via Endogenous Ca2+ Redistribution for Tumor Radiosensitization,” Advanced Materials (2019): 1805875. 10.1002/adma.202401222 38690593

[10] Q. Fu , R. Zhu , J. Song , H. Yang , and X. Chen , “Photoacoustic Imaging: Contrast Agents and Their Biomedical Applications,” Advanced Materials 31 (2019): 1805875. 10.1002/adma.201805875 30556205

[11] J. Weber , P. C. Beard , and S. A.‐O. Bohndiek , “Contrast Agents for Molecular Photoacoustic Imaging,” Nature Methods 13 (2016): 639–650, 10.1038/nmeth.3929. 27467727

[12] Q. Shen , L. Wang , X. Ruan , et al., “Stimuli‐Responsive Organic Near‐Infrared Photoacoustic Probes,” Advanced Functional Materials 33 (2023): 2300023, 10.1002/adfm.202300023.

[13] Y. Wu , F. Zeng , Y. Zhao , and S. Wu , “Emerging Contrast Agents for Multispectral Optoacoustic Imaging and their Biomedical Applications,” Chemical Society Reviews 50 (2021): 7924–7940, 10.1039/D1CS00358E. 34114588

[14] D. Lee , S. Beack , J. Yoo , et al., “In Vivo Photoacoustic Imaging of Livers Using Biodegradable Hyaluronic Acid‐Conjugated Silica Nanoparticles,” Advanced Functional Materials 28 (2018): 1800941.

[15] Z. Zhao , C. X. Swartchick , and J. Chan , “Targeted Contrast Agents and Activatable Probes for Photoacoustic Imaging of Cancer,” Chemical Society Reviews 51 (2022): 829–868, 10.1039/D0CS00771D. 35094040 PMC9549347

[16] Q. Miao , Y. Lyu , D. Ding , and K. Pu , “Photoacoustic Imaging: Semiconducting Oligomer Nanoparticles as an Activatable Photoacoustic Probe With Amplified Brightness for In Vivo Imaging of pH,” Advanced Materials 28 (2016): 3662–3668, 10.1002/adma.201670129. 27000431

[17] P. Cheng , W. Chen , S. Li , S. He , Q. Miao , and K. Pu , “Fluoro‐Photoacoustic Polymeric Renal Reporter for Real‐Time Dual Imaging of Acute Kidney Injury,” Advanced Materials 32 (2020): 1908530. 10.1002/adma.201908530 32141674

[18] Z. Chen , B. Yun , Y. Hou , et al., “NIR‐II Anti‐Stokes Luminescence Nanocrystals With 1710 nm Excitation for in Vivo Bioimaging,” Angewandte Chemie International Edition 64 (2025): 202416893, 10.1002/anie.202416893. 39382037

[19] L. Zeng , G. Ma , H. Xu , et al., “In Vivo Chemoselective Photoacoustic Imaging of Copper(II) in Plant and Animal Subjects,” Small 15 (2019): 1803866. 10.1002/smll.201803866 30645025

[20] M. Gifani , D. J. Eddins , H. Kosuge , et al., “Ultraselective Carbon Nanotubes for Photoacoustic Imaging of Inflamed Atherosclerotic Plaques,” Advanced Functional Materials 31 (2021): 2101005, 10.1002/adfm.202101005. 34733130 PMC8559995

[21] W. Qin , J. Huang , C. Yang , et al., “Protease‐Activatable Nanozyme With Photoacoustic and Tumor‐Enhanced Magnetic Resonance Imaging for Photothermal Ferroptosis Cancer Therapy,” Advanced Functional Materials 33 (2023): 2209748, 10.1002/adfm.202209748.

[22] A. De La Zerda , C. Zavaleta , S. Keren , et al., “Carbon Nanotubes as Photoacoustic Molecular Imaging Agents in Living Mice,” Nature Nanotechnology 3 (2008): 557–562, 10.1038/nnano.2008.231. PMC2562547 18772918

[23] Q. Chen , C. Liang , X. Sun , et al., “H_2_O_2_‐Responsive Liposomal Nanoprobe for Photoacoustic Inflammation Imaging and Tumor Theranostics via in Vivo Chromogenic Assay,” Proceedings of the National Academy of Sciences 114 (2017): 5343–5348, 10.1073/pnas.1701976114. PMC5448233 28484000

[24] J. Li , D. Ding , J. Wang , L. Huang , J. Zhan , and W. Lin , “An Activatable Photoacoustic Probe for Imaging Upregulation of Hydrogen Sulfide in Inflammation,” Sensors and Actuators B: Chemical 367 (2022): 132097, 10.1016/j.snb.2022.132097.

[25] S. Das , H. Mazumdar , K. R. Khondakar , and A. Kaushik , “Machine Learning Integrated Graphene Oxide‐Based Diagnostics, Drug Delivery, Analytical Approaches to Empower Cancer Diagnosis,” Biomedical Engineering Materials 3 (2025): 12117.

[26] Z. Tu , P. Timashev , J. Chen , and X.‐J. Liang , “Ferritin‐Based Drug Delivery System for Tumor Therapy,” Biomedical Engineering Materials 1 (2023): 12022.

[27] B. Wang , X. Hu , F. Sun , Z. Yang , and W. Huang , “Advanced Strategic Constructions of Diketopyrrolopyrrole Derivatives‐Based Organic Semiconducting Phototheranostics,” Interdisciplinary Medicine 1 (2023): 20220010, 10.1002/INMD.20220010.

[28] L. Zheng , R. Zhu , L. Chen , et al., “X‐Ray Sensitive High‐Z Metal Nanocrystals for Cancer Imaging and Therapy,” Nano Research 14 (2021): 3744–3755, 10.1007/s12274-021-3337-8.

[29] Z. Zhou , B. Guan , H. Xia , R. Zheng , and B. Xu , “Particle Radiotherapy in the Era of Radioimmunotherapy,” Cancer Letters 567 (2023): 216268, 10.1016/j.canlet.2023.216268. 37331583

[30] H. Wang , D. Lan , H. Cao , et al., “Self‐Propulsion of Biomimetic Nanomotors Promotes Diffusion and Convection Transport for Enhanced Radiotherapy in Solid Glioblastoma,” Journal of the American Chemical Society 147 (2025): 25072–25087, 10.1021/jacs.5c09121. 40587590

[31] Y. Zhang , X. Han , Y. Liu , S. Wang , X. Han , and C. Cheng , “Research Progress on Nano‐Sensitizers for Enhancing the Effects of Radiotherapy,” Materials Advances 3 (2022): 3709–3725, 10.1039/D2MA00094F.

[32] N. Ma , F. Wu , X. Zhang , et al., “Shape‐Dependent Radiosensitization Effect of Gold Nanostructures in Cancer Radiotherapy: Comparison of Gold Nanoparticles, Nanospikes, and Nanorods,” ACS Applied Materials & Interfaces 9 (2017): 13037–13048, 10.1021/acsami.7b01112. 28338323

[33] P. Liu , Z. Huang , Z. Chen , et al., “Silver Nanoparticles: A Novel Radiation Sensitizer for Glioma?,” Nanoscale 5 (2013): 11829–11836, 10.1039/c3nr01351k. 24126539

[34] Y. Ogawa , “Paradigm Shift in Radiation Biology/Radiation Oncology—Exploitation of the “H_2_O_2_ Effect” for Radiotherapy Using Low‐LET (Linear Energy Transfer) Radiation such as X‐rays and High‐Energy Electrons,” Cancers 8 (2016): 28. 26927177 10.3390/cancers8030028 PMC4810112

[35] Y. Zhang , M. Jeon , L. J. Rich , et al., “Non‐Invasive Multimodal Functional Imaging of the Intestine With Frozen Micellar Naphthalocyanines,” Nature Nanotechnology 9 (2014): 631–638, 10.1038/nnano.2014.130. PMC4130353 24997526

[36] X. Qin , C. Yang , H. Xu , et al., “Cell‐Derived Biogenetic Gold Nanoparticles for Sensitizing Radiotherapy and Boosting Immune Response Against Cancer,” Small 17 (2021): 2103984, 10.1002/smll.202103984. 34723421

[37] L. Chen , J. Zhang , L. Xu , et al., “Composition Tunability of Semiconductor Radiosensitizers for Low‐Dose X‐Ray Induced Photodynamic Therapy,” Journal of Nanobiotechnology 20 (2022): 293, 10.1186/s12951-022-01494-7. 35729553 PMC9210653

[38] Z. Arab‐Bafrani , E. Zabihi , S. M. Jafari , et al., “Enhanced Radiotherapy Efficacy of Breast Cancer Multi Cellular Tumor Spheroids Through in‐Situ Fabricated Chitosan‐Zinc Oxide Bio‐Nanocomposites as Radio‐Sensitizing Agents,” International Journal of Pharmaceutics 605 (2021): 120828, 10.1016/j.ijpharm.2021.120828. 34174360

[39] G. Song , L. Cheng , Y. Chao , K. Yang , and Z. Liu , “Emerging Nanotechnology and Advanced Materials for Cancer Radiation Therapy,” Advanced Materials 29 (2017): 1700996. 10.1002/adma.201700996 28643452

[40] Q. Liu , X. Ding , X. Xu , et al., “Tumor‐Targeted Hyaluronic Acid‐Based Oxidative Stress Nanoamplifier With ROS Generation and GSH Depletion for Antitumor Therapy,” International Journal of Biological Macromolecules 207 (2022): 771–783, 10.1016/j.ijbiomac.2022.03.139. 35351548

[41] Y. Li , W. Chen , Y. Qi , et al., “H_2_ S‐Scavenged and Activated Iron Oxide‐Hydroxide Nanospindles for MRI‐Guided Photothermal Therapy and Ferroptosis in Colon Cancer,” Small 16 (2020): 2001356, 10.1002/smll.202001356. 32789963

[42] Y. Yang , S. Guan , Z. Ou , W. Li , L. Yan , and B. Situ , “Advances in AI‐Based Cancer Cytopathology,” Interdisciplinary Medicine 1 (2023): 20230013, 10.1002/INMD.20230013.

[43] S. Wang , S. Yang , Z. Cui , et al., “In‐Situ Activation of CuAl‐LDH Nanosheets to Catalyze Bioorthogonal Chemistry in Vivo in Tumor Microenvironment for Precise Chemotherapy and Chemodynamic Therapy,” Chemical Engineering Journal 457 (2023): 141186, 10.1016/j.cej.2022.141186.

[44] E. Feng , Y. Liu , S. Lv , et al., “Fine‐Tuning Cu (II)‐Induced Self‐Assembly of Hydrophilic Cyanine Dyes for Enhanced Tumor Photothermal Therapy,” Advanced Functional Materials 32 (2022): 2209258, 10.1002/adfm.202209258.

[45] M. P. Ghosh , S. Datta , R. Sharma , K. Tanbir , M. Kar , and S. Mukherjee , “Copper Doped Nickel Ferrite Nanoparticles: Jahn‐Teller Distortion and its Effect on Microstructural, Magnetic and Electronic Properties,” Materials Science and Engineering: B 263 (2021): 114864, 10.1016/j.mseb.2020.114864.

[46] R. R. Gathje , J. Steuer , K. R. Steuer , R. Nicholes , and K. R. Nicholes , “Stability Studies on Indocyanine Green Dye,” Journal of Applied Physiology 29 (1970): 181–185, 10.1152/jappl.1970.29.2.181. 4913806

