# Supplementary material for: In Situ Synthesis of a Tumor Microenvironment‐Activated Radiosensitizing Cu2‐xS/LDH Probe for Photoacoustic Imaging‐Guided Radiotherapy
Source: Adv Sci (Weinh). 2026 Jan 25;13(19):e22370. doi: 10.1002/advs.202522370 (PMC13045243; doi:10.1002/advs.202522370)
Supplement: Supplementary file 1 — Supporting File: advs74027‐sup‐0001‐SuppMat.docx. [file ADVS-13-e22370-s001.docx]

Supporting Information

***In Situ* Synthesis of a Tumor Microenvironment-Activated Radiosensitizing Cu₂₋ₓS/LDH Probe for Photoacoustic Imaging-Guided Radiotherapy**

*Kang Zhu^#^, Qiaoqiao Wei^#^, Ying Wu*, Qingqing Li, Luntao Liu, Chunxiang Mo, Chuqiao Liu, Lichao Su, Jibin Song**

K. Zhu, Q.Q. Wei, Y. Wu, C.X. Mo, C.Q. Liu, Prof. J. B. Song

State Key Laboratory of Chemical Resource Engineering, College of Chemistry, Beijing University of Chemical Technology, Beijing 100029, China.

Email: [jibin.song@buct.edu.cn](mailto:jibin.song@buct.edu.cn)

Dr. Q.Q. Li, Dr. L.T. Liu, Dr. L.C. Su

College of Chemistry, Fuzhou University, Fuzhou 350108, P. R. China.

***Materials:***

Sodium hydroxide (NaOH), Copper nitrate (Cu(NO_3_)_2_), Aluminum nitrate (Al(NO_3_)_3_), Sodium Nitrate (NaNO_3_) and Sodium Sulfide (Na_2_S) were provided by Sigma-Aldrich (St. Louis, USA). Methanol (CH_3_OH) and hydrogen peroxide (H_2_O_2_) were acquired from Sinopharm Chemical Reagent Beijing Co., Ltd (Beijing, China). Fetal bovine serum (FBS) and Roswell Park Memorial Institute 1640 Medium (RPMI 1640) were obtained from Gibco Life Technologies (Grand Island, USA). Cell Counting Kit-8 (CCK-8), 2′,7′-dichlorofluorescin diacetate (DCFH-DA), Annexin V-FITC/PI apoptosis detection Kit, and Calcein-AM/ PI live/dead assay kit were supplied from Meilunbo (Dalian, China). Ultrapure water was used for all synthesis experiments. Commercially available chemical reagents were used without further purification.

***Characterizations:***

Transmission electron microscopy (TEM) images were recorded on an FEI Tecnai F20 transmission electron microscope (Thermo Fisher Scientific, USA). Scanning electron microscopy (SEM) images were acquired on SEM 230 (FEI, Czech Republic). Malvern-Zetasizer Nano ZS Instruments (Malvern, UK) was used to measure the hydrodynamic diameter and zeta potential. The Ultraviolet-visible near-infrared (UV-vis-NIR) absorption spectra were performed using a UH4150 spectrophotometer (Hitachinaka, Japan). X-ray photoelectron spectra (XPS) results were obtained by Thermo ESCALAB 250Xi X-ray photoelectron spectrometer (Thermo Fisher Scientific, USA). X-ray diffraction (XRD) patterns were measured using an Ultima IV 285 X-ray powder diffractometer (Rigaku Co., Japan). Confocal laser scanning microscopy (CLSM) images of cells were obtained by a Nikon A1 confocal fluorescence microscope (Nikon, Japan). Flow cytometry analysis was carried out on a CytoFLEX instrument (Beckman Colter, USA). Cell viability was monitored on the Infinite M200 Pro NanoQuant microplate reader (TECAN, Switzerland). Photoacoustic (PA) images were obtained by applying the Vevo LAZR-X PA imaging system (Visual-Sonics Co. Ltd, Canada).

***Methods***

***In vitro* PA imaging of CAL-IR nanoparticles on CT26 cells.** PA imaging experiments were performed using several control groups to investigate the response of the composite nanoprobes to H_2_S in CT26 cells. CT26 cells were cultured overnight at 37 °C under 5% CO_2_. The culture medium was discarded, and the cells were washed once with PBS (pH 7.4). Subsequently, CT26 cells were divided into four groups (each in triplicate) and differently treated as follows: I) Control group (only CT26 cells); II) Material group (CT26 cells incubated with CAL-IR probes for 12 hours); III) Inhibition group (CT26 cells pre-treated with zinc chloride (ZnCl_2_) solution for 5 minutes to reduce H_2_S levels, followed by incubation with CAL-IR probes for 12 hours); IV) Promotion group (CT26 cells pre-treated with L-cysteine (L-Cys) for 1 hour to increase intracellular H_2_S levels, followed by incubation with CAL-IR probes for 12 hours)., the CT26 cells from all four groups were detached from the culture dishes after the incubation time using trypsin, centrifuged at 1000 rpm for 3 minutes, collected, and resuspended in phosphate buffered saline (PBS, pH 7.4). The PA images of the cell suspension at 835 nm and 1250 nm were detected and recorded using a PA imaging system, followed by data analysis.

**Flow cytometry analysis**. One mL CT26 cell suspension, resuspended in 1640 complete medium after digestion, was seeded into a 6-well plate and incubated for 24 hours at 37 °C under 5% CO_2_. Cells were divided into four groups: PBS, X-ray (4 Gy), CAL-IR (80 μg/mL), and X-ray (4 Gy) + CAL-IR (80 μg/mL) and incubated for an additional 10 hours. Cells were treated with Annexin V-FITC/PI apoptosis according to the manufacturer instruction of the same detection kit and analyzed by flow cytometry.

***In vivo* NIR-I and NIR-II PA imaging of H_2_S in subcutaneous tumor.** Six to eight-week-old female Balb/c mice, weighing between 18 and 20 grams, were purchased at Beijing Vital River Laboratory Animal Technology Co., Ltd. All animal experiments in this study were carried out in compliance with the protocol approved by the Institutional Animal Ethics Committee of Fujian Medical University (Ministry of Science and Technology of China, 2013 IACUC-2013-012). Log-phase CT26 cells were digested and resuspended in PBS to a concentration of approximately 1×10^7^ cells/mL, then subcutaneously injected into the hind limbs of the mice to establish a tumor model. The composite nanoprobe CAL-IR (100 μL, 2 mg/mL) was administered through tail vein injection to allow its accumulation in the tumor.

The mice were pre-anesthetized during the testing process using a gas anesthesia machine filled with isoflurane in a transparent anesthesia chamber. Subsequently, the mice were placed in the detection area, and PA images of the tumor sites were collected at various time points (0, 9, 12, 15, 18, 21, and 24 hours) using the Vevo LAZR-X 3100 PA imaging system at wavelengths of 835 nm and 1250 nm. The resulting PA images and their corresponding quantitative average PA signals were analyzed using VevoLAB software.

**Statistical Analysis.** All data were presented as mean ± SD (standard deviation). Comparison of the data was conducted with a t-test. Differences were regarded as statistically significant (*P < 0.05, **P < 0.01, ***P < 0.001).


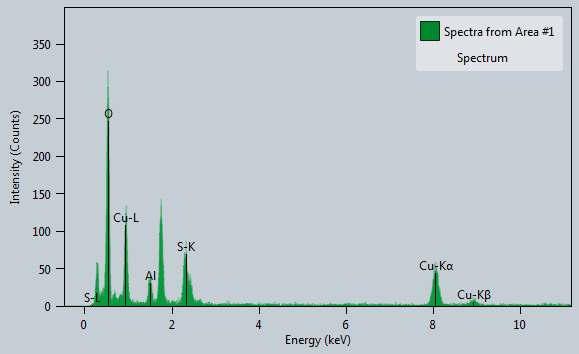


**Figure S1.** Element surface scanning (EDS) analysis of Cu-LDH.


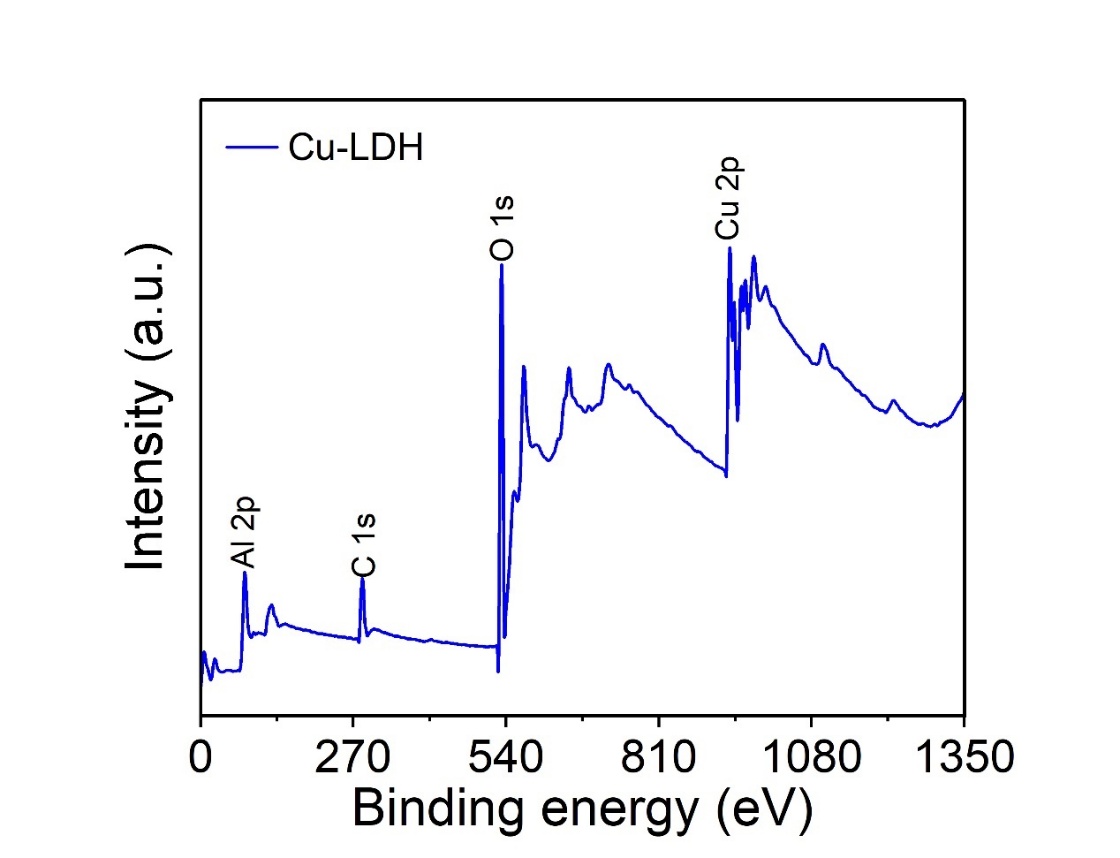


**Figure S2.** X-ray photoelectron spectroscopy (XPS) analysis of Cu-LDH.


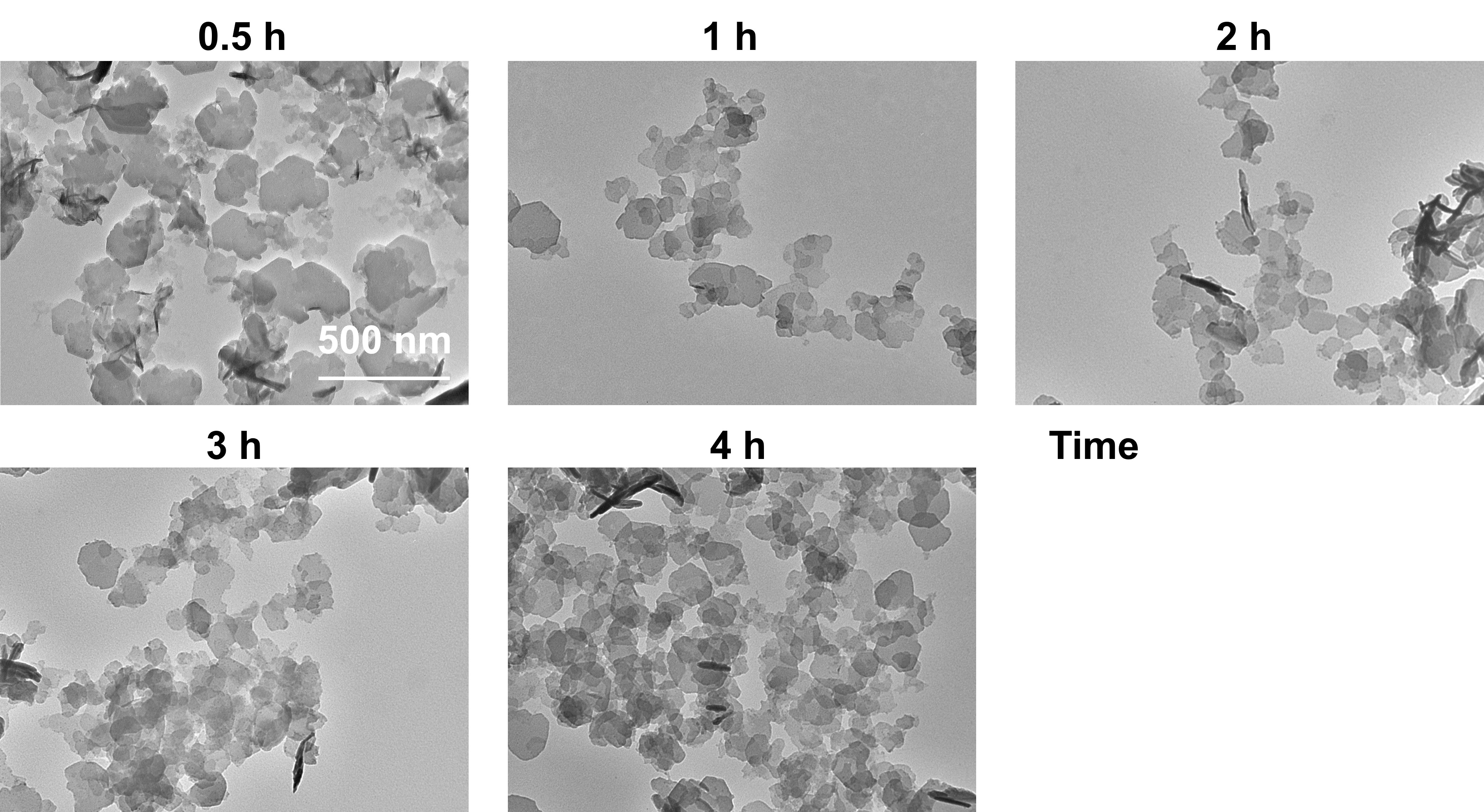


**Figure S3.** TEM image of the obtained Cu_2-x_S/CAL-IR under different response times.


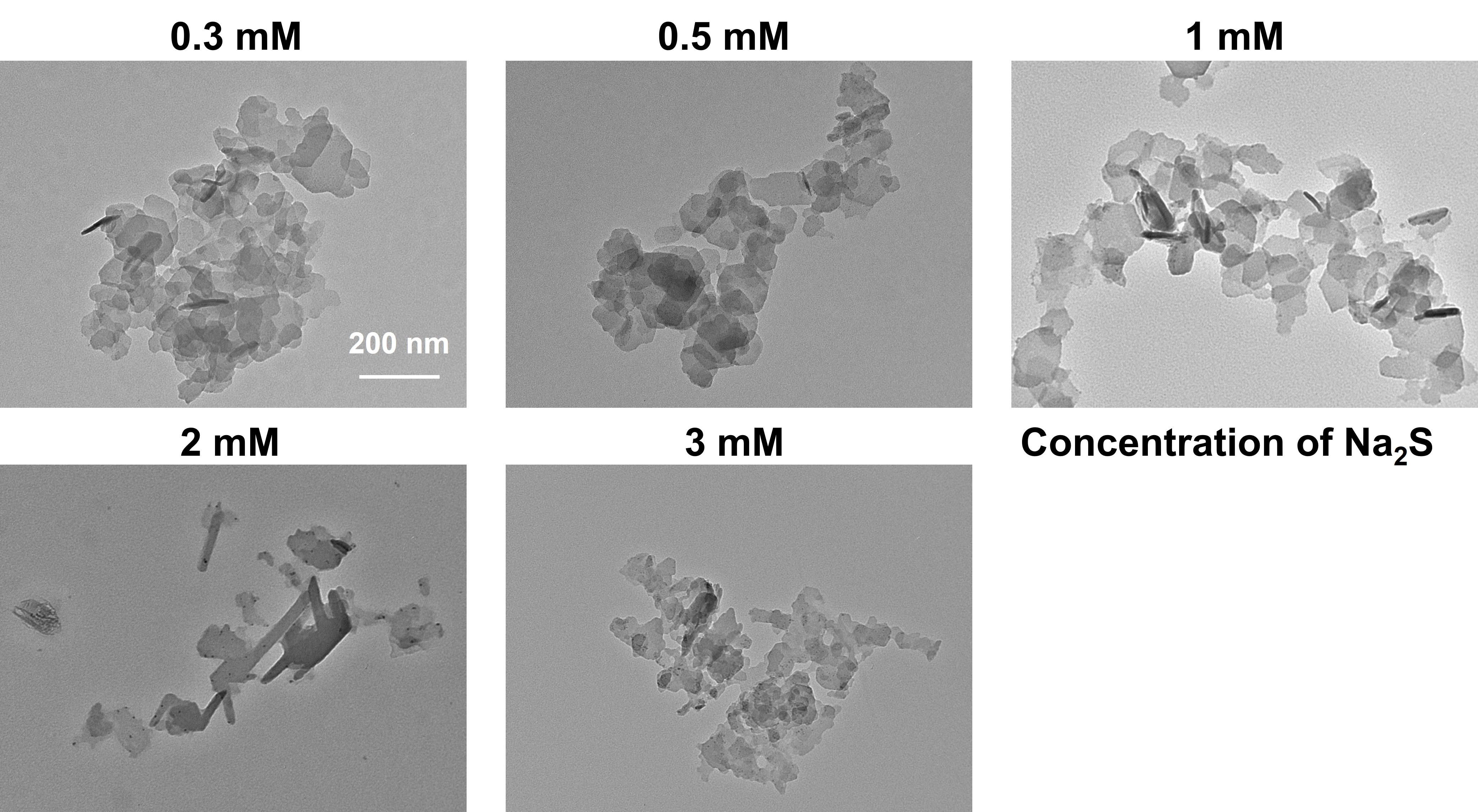


**Figure S4.** TEM image of the obtained Cu_2-x_S/CAL-IR at different concentrations of Na_2_S.


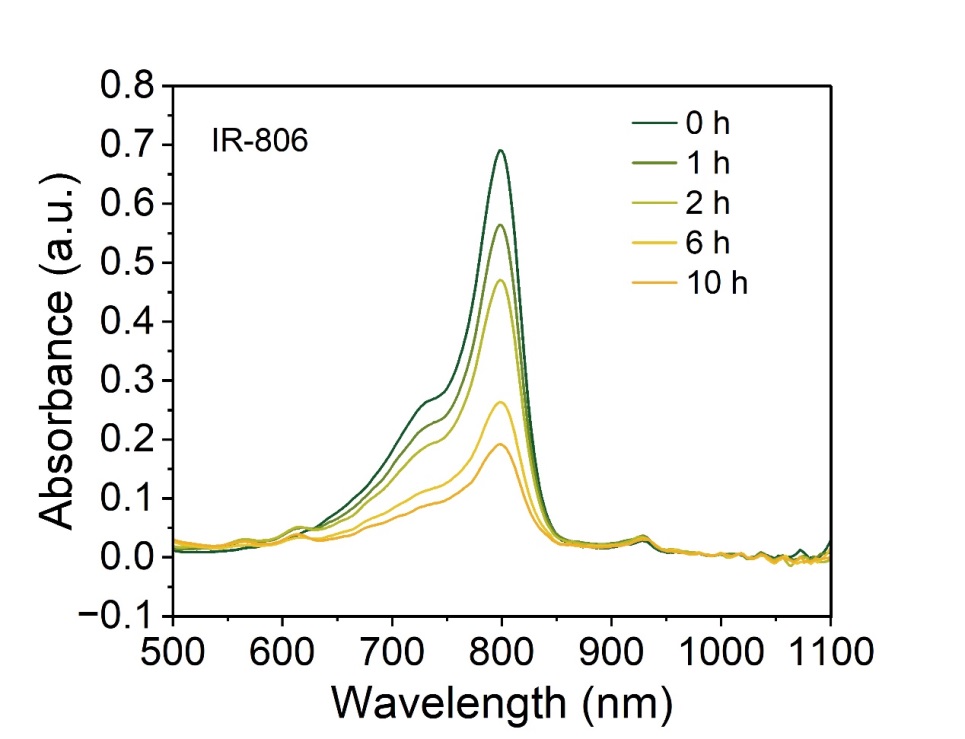


**Figure S5.** UV-visible absorption spectra of IR-806 over time.


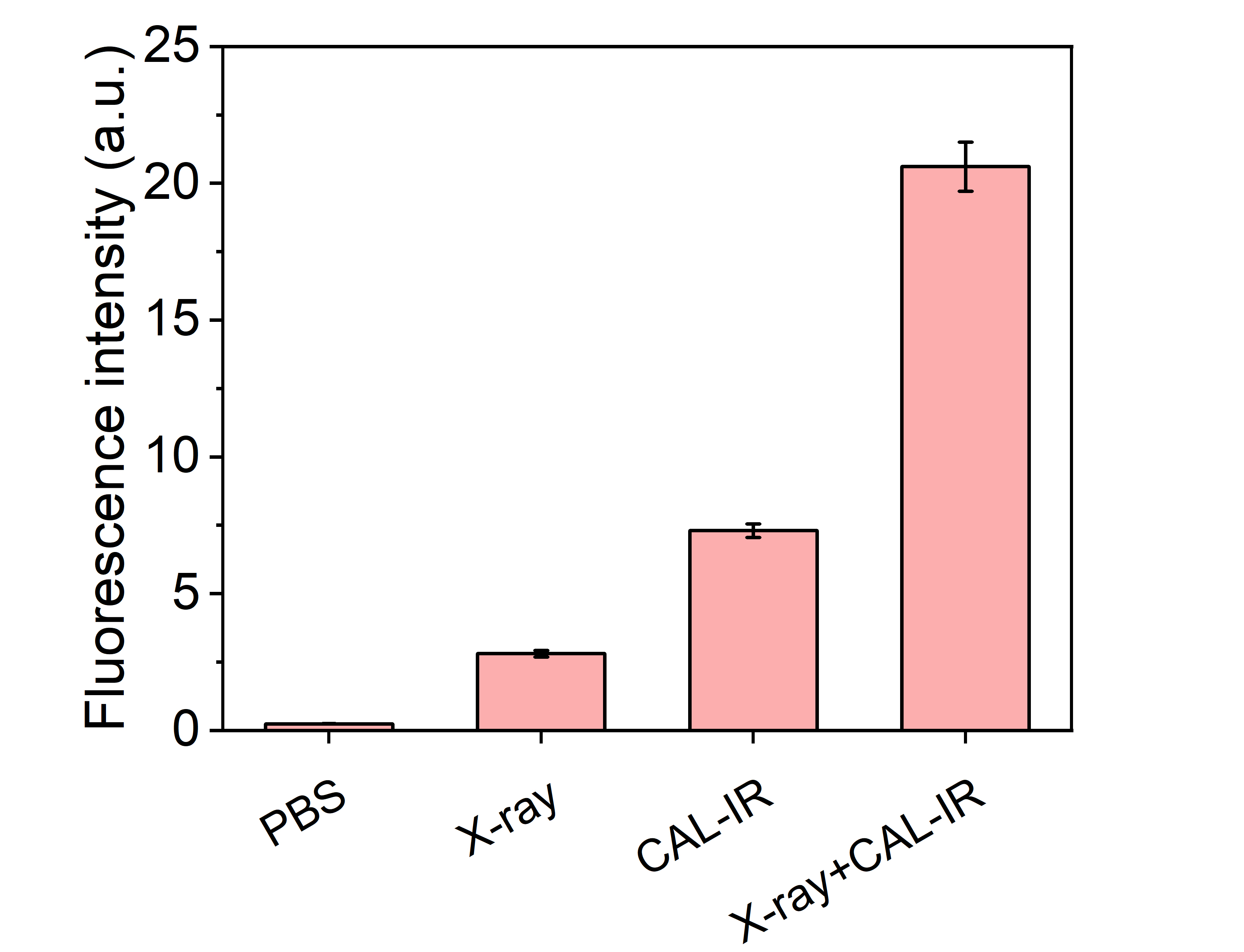


**Figure S6.** Quantitative analysis of fluorescence intensity of CT26 cells treated with different methods in the ROS detection process.


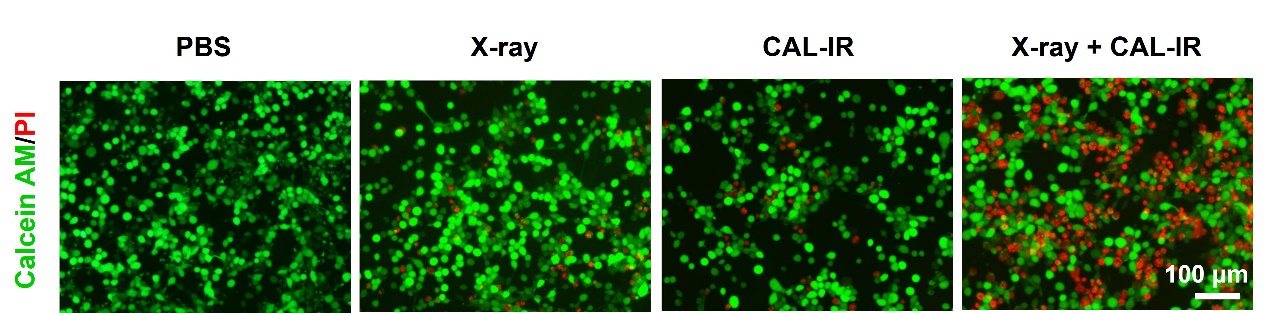


**Figure S7.** The calcein AM and propidium iodide (PI) double staining assay of different treatment groups.


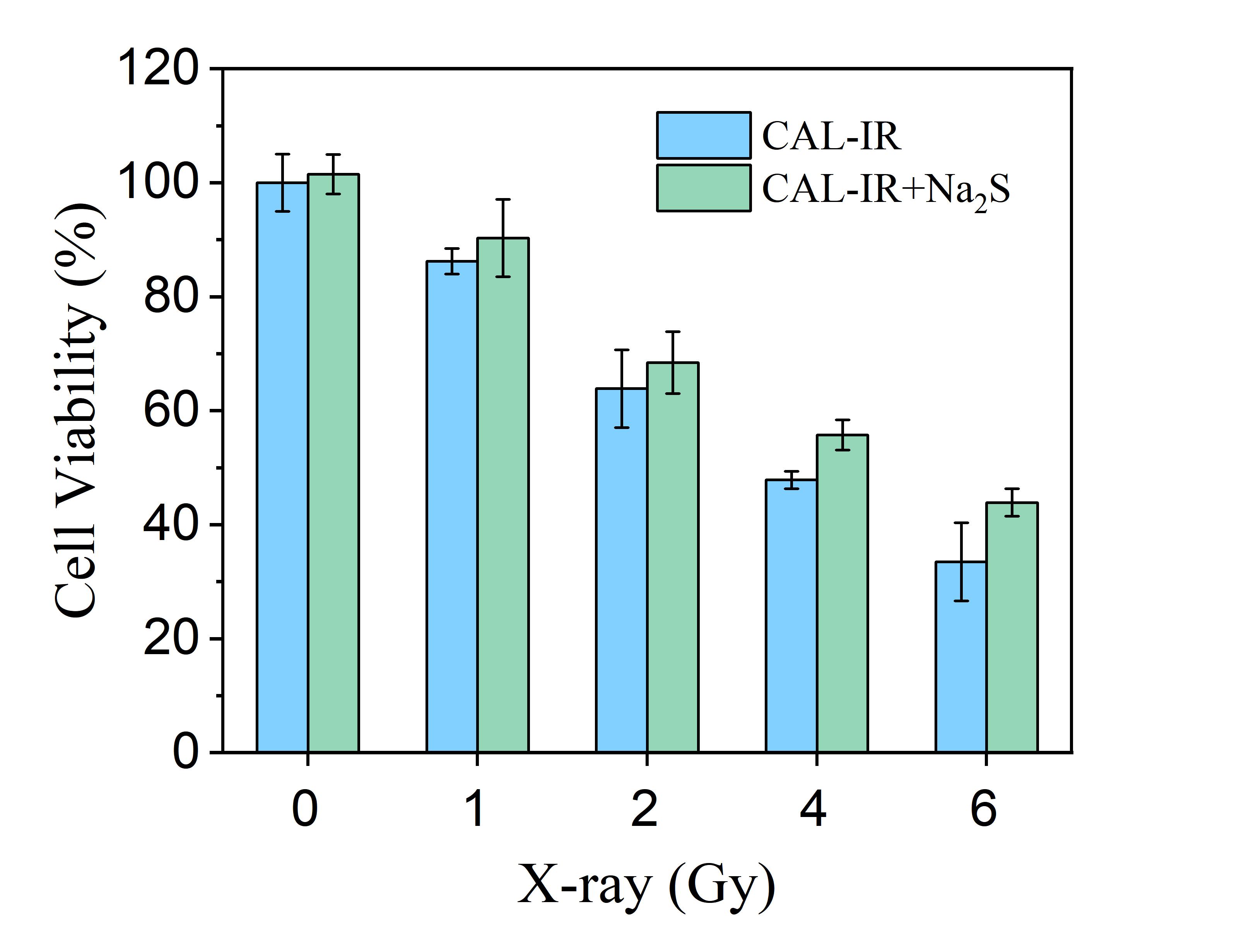


**Figure S8.** The Viability of CT26 cells treated with CAL-IR/CAL-IR+Na₂S and irradiated with different doses of X-ray.


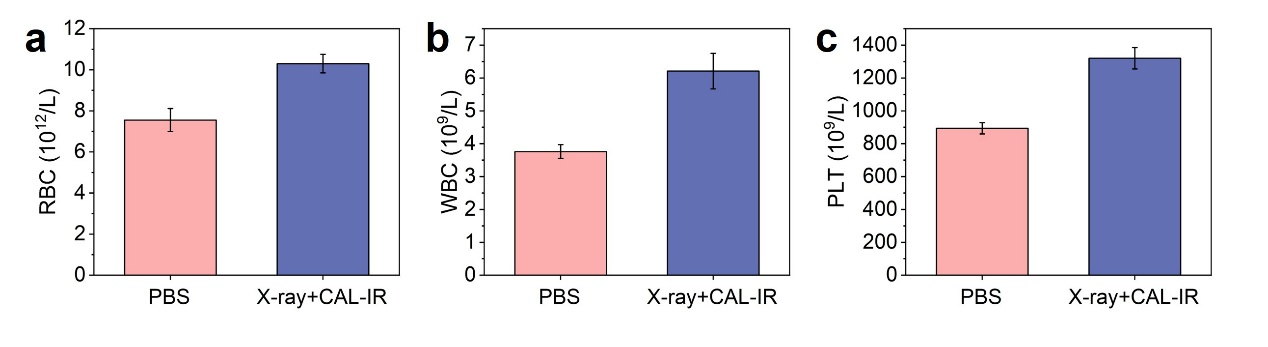


**Figure S9.** The levels of (a) RBC, (b) WBC, and (c) PLT in routine blood tests.
